# Clinical Significance of Preoperative Thrombin Time in Patients with Esophageal Squamous Cell Carcinoma following Surgical Resection

**DOI:** 10.1371/journal.pone.0140323

**Published:** 2015-10-15

**Authors:** Xiao-Hui Li, Xue-Ping Wang, Wen-Shen Gu, Jian-Hua Lin, Hao Huang, Ting Kang, Lin Zhang, Hao Chen, Xin Zheng

**Affiliations:** 1 State Key Laboratory of Oncology in South China, Collaborative Innovation Center for Cancer Medicine, Guangzhou, China; 2 Department of Clinical Laboratory Medicine, Sun Yat-Sen University Cancer Center, Guangzhou, China; 3 Department of Clinical Laboratory Medicine, Sun Yat-Sen Memorial Hospital, Guangzhou, China; 4 Department of Clinical Laboratory Medicine, The First Affiliated Hospital of Sun Yat-Sen University, Guangzhou, China; 5 Guangdong Esophageal Cancer Institute, Guangzhou, China; INRS, CANADA

## Abstract

**Background:**

Noninvasive tools for the prognosis of ESCC are urgently needed. To this end, serum coagulation tests have been researched in some cancers, but the prognostic value of the TT in ESCC has not been described.

**Methods:**

The levels of pre-treatment serum coagulation markers (including the PT, APTT, PTA, INR, fibrinogen level, TT and PLT) were retrospectively analyzed in 204 patients with ESCC who underwent surgical resection at our department and in 200 healthy controls, and the two groups were compared. The prognostic significance of the coagulation tests was then determined with univariate and multivariate cox hazard analyses in patients with ESCC.

**Results:**

Compared with those in normal controls, the PT, APTT, and fibrinogen levels were significantly increased, whereas the TT values significantly decreased in the 204 ESCC patients. The TT directly correlated with the 5-year survival rate, not only in the entire ESCC cohort (*p* = 0.023) but also in the subgroups stratified according to the T and N classifications and metastasis. Conversely, the other tests were not independent prognostic factors for ESCC. Of the clotting markers, the TT inversely correlated with the fibrinogen level (*p* = 0.005).

**Conclusions:**

The 5-year survival was shorter in ESCC patients exhibiting decreased pre-treatment TT values. Thus, the serum TT may be a clinical prognostic factor for ESCC patients.

## Introduction

Esophageal squamous cell carcinoma (ESCC) is the major pathological type of esophageal cancer and one of the most common malignancies worldwide. Furthermore, it is the fourth most frequent cause of cancer-related death in China [1–2]. Although advanced diagnostic tools, surgical techniques and therapy are available for patients with ESCC, the 5-year survival rate remains low [3–4]. Thus, a more effective biomarker to recognize the biological characteristics of ESCC patients needs to be identified in order to guide individualized treatment.

The association between cancer and thrombosis is well recognized, and almost all types of cancer are associated with an activation of coagulation, even in the absence of thrombosis [5–8]. Patients with cancer are at a 4- to 6-fold increased risk of developing venous thromboembolism (VTE) [9]. Importantly, cancer-induced hemostatic activity has been shown to promote tumor progression and dissemination, the inflammatory cell response, tumor angiogenesis, and metastasis [10–12]. In fact, abnormal coagulation parameters that represent active coagulation and fibrinolytic systems have been associated with tumor progression and decreased overall survival (OS). Some researchers reported that increases in the prothrombin time (PT) and international normalized ratio (INR) are associated with decreased survival in lung cancer patients [13]. Moreover, high levels of circulating biomarkers, such as fibrinogen, fibrin(ogen) split products and D-dimer, are associated with decreased overall survival (OS) in breast cancer, colorectal cancer and melanoma [14–16]. High D-dimer levels and hyperfibrinogenemia were also identified as independent prognostic predictors in pancreatic cancer and gallbladder cancer, respectively [17–20]. The thrombin time (TT) is one of the most common coagulation tests used in the laboratory, but its prognostic value in cancer has rarely been studied. Moreover, few studies have systematically investigated the relationship between ESCC and coagulation abnormalities, and the prognostic value of the TT consequently remains unclear.

The aim of the present study was to assess the clinical and prognostic significance of various plasma coagulation tests in ESCC patients and to delineate the correlation of these coagulation tests with other clinical variables.

## Subjects and Methods

### Subjects

This study examined 204 consecutive patients (145 men and 59 women; ages 36–79 years, median 59 years) who received histologically confirmed diagnoses of ESCC between January 2007 and May 2008 at the Sun Yat-sen University Cancer Center in Guangzhou, China. Patients with one of the following conditions were excluded: (1) patients younger than 18 years of age; (2) patients who received antitumor treatment or surgery before enrolling in this study; (3) patients who regularly received procoagulant or anticoagulant therapy or received blood transfusions within 1 month of study onset; (4) patients who were diagnosed with VTE, pulmonary embolism, or disseminated intravascular coagulation (DIC) within 1 month of study onset or during the subsequent treatment. Using the patients’ medical records, the grade of tumor differentiation and stage were classified based on the 6th edition of the AJCC/UICC TNM system. In our cohort, all 204 patients underwent surgical resection. Specifically, 65.7% (134/204) patients underwent tumor resection without receiving any treatment, and 34.3% (70/204) patients received other comprehensive therapy. Furthermore, 27.5% (56/204) of patients received chemotherapy after surgery: 5 patients received radiation and 9 patients received radiation and chemotherapy after surgery. Only one patient received radiation and chemotherapy prior to surgery (stage IV). None of the stage I patients, 17.5% (17/97) of stage II patients and 57.5% (52/92) of stage III and IV patients received radiation and/or chemotherapy after surgery. The most common chemotherapeutics were capecitabine, floxuridine and ethyleniminoquinonum.

A total of 200 healthy participants (142 men and 58 women; ages 36–77 years, median 58years) free of tumors and coagulation disorders (such as VTE, pulmonary embolism, or disseminated intravascular coagulation) were recruited from the physical examination department at the Sun Yat-Sen University Cancer Center. Patients younger than 18 years or older than 80 years of age were excluded.

Detailed clinical and pathological information, including demographic data, smoking status, pathological tumor, node, metastasis stage and overall survival data, were available for all patients. The overall patient survival, defined as the time from surgery to death or last follow up, whichever came first, was used as a measure of prognosis.

### Laboratory Measurements

Venous blood samples were collected in tubes containing sodium citrate prior to the initiation of any treatment to measure coagulation parameters. The samples were immediately centrifuged and studied within 2 h of processing according to the instructions from the manufacturer. The PT, APTT, fibrinogen and TT were measured using a Sysmex CA-7000 automatic coagulation analyzer (Sysmex Corporation, Kobe, Japan). Commercially available reagents (Siemens AG, Munich, Germany) provided by the kinetic nephelometric detection system using a Diagon Dia-Timer 4 (Diagon Ltd, Budapest, Hungary) were employed to measure the PT, APTT, TT and fibrinogen level. The prothrombin activity (PTA) reflects the calibration of PT, and PTA and INR were calculated based on the PT. The platelet count (PLT) was derived from the blood count using a Sysmex XE-5000 automatic blood-cell counter (Sysmex Corporation, Kobe, Japan). Prior to use of these patients’ sera, written informed consent was obtained from each of the participants, and the experiment was approved by the Institute Research Ethics Committee of Cancer Center of Sun Yat-Sen University in Guangzhou, China. Data made available to all interested researchers upon request. E-mail: zhengxin@sysucc.org.cn; chenhao@sysucc.org.cn

### Statistical Analysis

The results are reported either as the mean or median values depending on the type of distribution. Continuous variables (INR, PTA, APTT, fibrinogen and PLT) were categorized using median values as cut-off points except for TT and PT, which were based on the mean±SD. The correlation between coagulation tests and clinical characteristics was assessed using the Mann-Whitney U test except for TT and PT, which were compared using independent t-tests and χ^2^ tests. The differences between ESCC patients and healthy donors were compared using the unpaired Student’s t-test. Univariate and multivariate analyses of clinical variables were performed using Cox proportional hazards regression models. The results of this survey were analyzed using the Kaplan-Meier survival curves with the log-rank test. P values < 0.05 were considered to indicate significant differences. All reported P values are two sided, and all statistical tests were performed with the SPSS 17.0 for Windows software (SPSS, Chicago, IL, USA).

## Results

### Comparison of coagulation tests between patients and healthy controls

**Fig 1:**
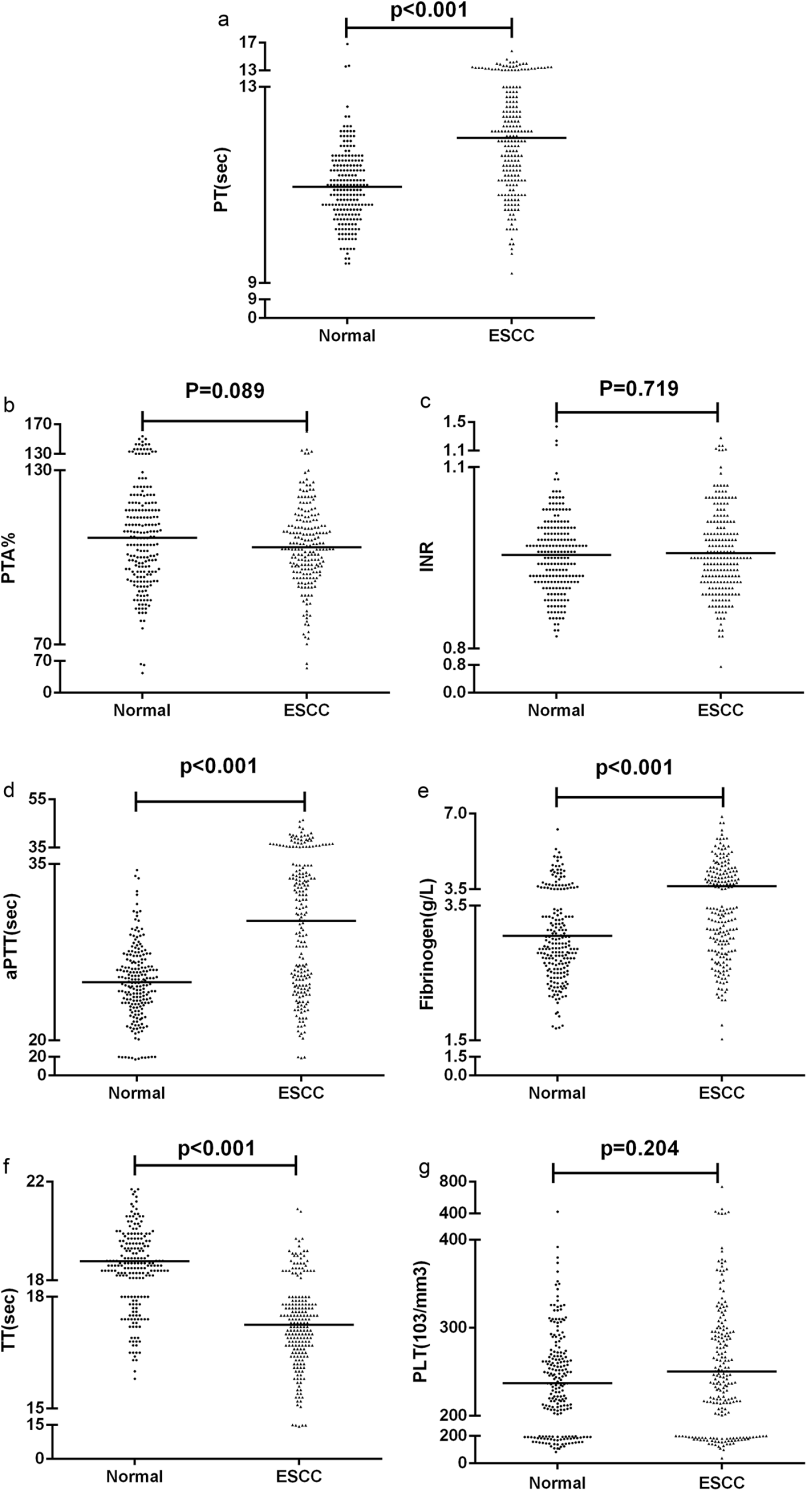
Pre-treatment serum levels of coagulation markers in patients with ESCC and in healthy controls. Pre-treatment serum levels of PT (a), PTA (b), INR (c), APTT (d), fibrinogen (e), TT (f), PLT (g) in ESCC and healthy controls. Each dot represents the level of one patient or one control, and the line in the graph indicates the median value.

To investigate whether coagulation abnormalities occur in ESCC, the coagulation tests results of healthy controls (n = 200) and ESCC patients (n = 204) were compared using the non-parametric test (Table 1). The plasma level of several coagulation markers, i.e., PT, APTT, fibrinogen and TT, revealed significant differences between the patient and control group (*p* < 0.001 for all variables). However, the PTA (*p* = 0.089), INR (*p* = 0.719) and PLT (*p* = 0.204) did not differ between these groups (Fig 1). Overall, the PT, APTT, fibrinogen, PLT levels were higher and the TT and PTA were lower in the ESCC patients than in the control group.

**Table 1:** The serum coagulation tests results in patients with ESCC and healthy controls.

	Patients (n = 204)		Control s(n = 200)		
Coagulation tests	Median	Range	Median	Range	p value
PT (sec)	12.00	9.2–15.8	10.90	9.4–16.8	<0.001
PTA (%)	102.90	54.6–160.8	106.80	43.8–154.1	0.09
INR	0.95	0.75–1.28	0.95	0.82–1.44	0.72
APTT (sec)	30.70	19.0–46.5	24.90	18.2–34.9	<0.001
fibrinogen (g/L)	3.44	1.52–6.87	2.89	1.68–6.28	<0.001
TT (sec)	17.20	14.4–20.9	18.60	15.8–24.2	<0.001
PLT (103/mm3)	238.50	37.4–740.0	237.00	83.0–424.0	0.20

### Coagulation tests and their correlations with clinical characteristics

The relationships between the clotting markers and clinical characteristics of the patients are summarized in Table 2. Specifically, patients classified as T1-T2 exhibited a significantly higher TT than patients classified as T3-T4 (*p* = 0.019). Moreover, the fibrinogen level significantly correlated histological differentiation, T classification, N classification and TNM stage (*p* = 0.012, *p* = 0.006, *p* = 0.012, and *p* = 0.004, respectively), suggesting that patients with moderate to advanced disease exhibit higher fibrinogen levels than patients with early-stage disease. Furthermore, 30.9% (63/204) of patients exhibited mild to moderate hyperfibrinogenemia (cut-off value 4.0 g/L). In addition, elevated PLT counts were associated with age lower than 59 (248000/mm^3^ vs 231000/mm^3^, *p* = 0.043), and 20.1% (41/204) of patients exhibited mild to moderate thrombocytosis (cut-off value 300000/mm^3^). However, none of the clinical characteristics significantly correlated with the PT, INR, PTA% or APTT.

**Table 2:** Main Clinical Characteristics of Patients Group According to Coagulation Parameter Levels.

		Coagulation Parameters (Median and Range)						
Variables	N(%)	PT(sec)	PTA(%)	INR	APTT (sec)	fibrinogen (g/L)	TT (sec)	PLT (10^3^/mm^3^)
Gender								
Male	145.00	11.9	102.8	0.95	30.3	3.46	17.1	237
	(71.10)	(9.2–15.8)	(54.6–160.8)	(0.75–1.28)	(1 9.9–46.5)	(1.52–6.57)	(14.4–20.9)	(37.4–740.0)
Female	59.00	12	103	0.94	30.8	3.41	17.3	247
	(28.90)	(9.8–14.6)	(64.5–131.0)	(0.83–1.17)	(19.0–40.6)	(2.21–6.87)	(14.9–19.1)	(98.0–422.0)
		*p* = 0.575	*p* = 0.999	*p* = 0.982	*p* = 0.468	*p* = 0.276	*p* = 0.535	*p* = 0.945
Age								
< 59 years	101.00	11.9	105	0.94	30.8	3.39	17	**248**
	(49.50)	(9.8–14.6)	(70.2–131.0)	(0.83–1.17)	(19.0–45.9)	(1.52–6.57)	(14.4–20.9)	**(37.4–740.0)**
≥59 years	103.00	12.1	101.1	0.95	30.5	3.52	17.4	**231**
	(50.50)	(9.2–15.8)	(54.6–160.8)	(0.75–1.28)	(19.9–46.5)	(1.73–6.87)	(14.9–19.6)	**(98.0–391.0)**
		*p* = 0.293	*p* = 0.131	*p* = 0.183	*p* = 0.943	*p* = 0.624	*p* = 0.308	***p* = *0*.*043***
Tobacco history								
No	76.00	12.1	102.9	0.95	31	3.32	17.5	242.5
	(37.30)	(9.2–14.6)	(64.5–160.8)	(0.75–1.17)	(19.0–40.6)	(1.73–6.87)	(14.9–19.7)	(98.0–422.0)
Yes	128.00	11.9	102.9	0.95	30.1	3.48	17	238
	(62.70)	(9.6–15.8)	(54.6–136.3)	(0.82–1.28)	(19.9–46.5)	(1.52–6.57)	(14.0–20.9)	(37.4–740.0)
		*p* = 0.483	*p* = 1.000	*p* = 0.696	*p* = 0.619	*p* = 0.183	*p* = 0.479	*p* = 0.944
Histological differentiation*								
Differentiated	140.00	12.1	103	0.95	31	**3.53**	17.2	242
	(68.60)	(9.2–14.6)	(64.5–160.8)	(0.75–1.17)	(19.0–46.5)	**(2.10–6.87)**	(14.9–20.9)	(37.4–740.0)
Undifferentiated	64.00	11.7	102.8	0.95	29.5	**3.18**	17.3	237
	(31.40)	(9.9–15.8)	(54.6–135.0)	(0.84–1.28)	(27.0–41.5)	**(1.52–6.24)**	(14.4–20.8)	(123.0–426.0)
		*p* = 0.500	*p* = 0.925	*p* = 0.413	*p* = 0.883	***p* = *0*.*012***	*p* = 0.890	*p* = 0.966
T classification								
T1-2	55.00	12.1	104	0.96	29.9	**3.16**	**17.5**	238
	(27.00)	(9.2–14.6)	(64.5–160.8)	(0.75–1.17)	(19.0–46.5)	**(1.52–6.57)**	**(15.2–20.9)**	(141.0–458.0)
T3-4	149.00	11.9	102.8	0.94	30.8	**3.49**	**17.1**	239
	(73.00)	(9.6–15.8)	(54.6–136.3)	(0.82–1.28)	(19.7–45.9)	**(1.73–6.87)**	**(14.4–19.7)**	(37.4–740.0)
		*p* = 0.630	*p* = 0.441	*p* = 0.249	*p* = 0.545	***p* = *0*.*006***	***p* = *0*.*019***	*p* = 0.687
N classification								
No	108.00	11.9	104.4	0.95	30.8	**3.39**	17.1	238.5
	(52.90)	(9.2–14.6)	(64.5–160.8)	(0.75–1.17)	(19.7–46.5)	**(1.52–6.57)**	(14.4–20.9)	(37.4–458.0)
N1-2	96.00	12	102.4	0.95	30.5	**3.5**	17.4	238.5
	(47.10)	(9.7–15.8)	(54.6–135.0)	(0.82–1.28)	(19.0–45.9)	**(2.35–6.87)**	(15.0–20.8)	(98.0–740.0)
		*p* = 0.567	*p* = 0.392	*p* = 0.531	*p* = 0.989	***p = 0*.*012***	*p* = 0.093	*p* = 0.652
Metastasis								
M0	190.00	11.9	102.9	0.95	30.6	3.42	17.2	237
	(93.10)	(9.2–15.8)	(54.6–160.8)	(0.75–1.28)	(19.0–46.5)	(1.52–6.87)	(14.4–20.9)	(37.4–740.0)
M1	14.00	12.2	103.7	0.95	32.6	3.89	17.3	251.5
	(6.90)	(10.1–14.2)	(72.2–135.0)	(0.84–1.13)	(21.2–40.2)	(2.54–6.57)	(15.1–19.4)	(110.0–376.0)
		*p* = 0.472	*p* = 0.958	*p* = 0.901	*p* = 0.718	*p* = 0.107	*p* = 0.426	*p* = 0.575
TNM stage								
I-II	112.00	11.9	104.7	0.95	30.4	**3.39**	17.2	240.5
	(54.90)	(9.2–14.6)	(64.5–160.8)	(0.75–1.17)	(19.0–46.5)	**(1.52–5.40)**	(14.4–20.9)	(128.0–458.0)
III-IV	92.00	12	101.1	0.95	31.3	**3.6**	17.3	237
	(45.10)	(9.7–15.8)	(54.6–135.0)	(0.82–1.28)	(20.2–45.9)	**(2.14–6.87)**	(15.0–19.7)	(37.4–740.0)
		*p* = 0.260	*p* = 0.314	*p* = 0.454	*p* = 0.575	***p* = *0*.*004***	*p* = 0.532	*p* = 0.835

Bold italics indicate significant differences (*p* < 0.05).

*Moderately and well differentiated histologic types were classified as differentiated carcinoma; Low levels of differentiation were defined as undifferentiated carcinoma.

### Survival analysis

In the entire cohort, the median survival of all patients was 43 months (95%CI 40.8–49.0 months). At the end of observation, 62.7% (128/204) patients had died due to disease-related or unrelated factors.

To evaluate the prognostic value of clotting tests in ESCC, the demographic data, clinicopathological features, smoking status and clotting markers were evaluated using univariate and multivariate Cox regression models (Table 3). The univariate analysis revealed that T classification (HR = 2.288; 95%CI: 1.412–3.709; *p* = 0.001), N classification (HR = 2.158; 95%CI: 1.491–3.124; *p* < 0.001), metastasis (HR = 2.543; 95%CI: 1.393–4.460; *p* = 0.002), TNM stage (HR = 2.108; 95%CI: 1.459–3.046; *p* < 0.001) and TT (HR = 0.658; 95%CI: 0.457–0.948; *p* = 0.025) were significantly associated with ESCC survival. A multivariate analysis was carried out based on TNM stage, TT, age, and gender to determine the utility of these factors as independent prognostic factors for survival. To eliminate the influence of collinearity, we excluded the factors of T classification, N classification, and metastasis in the multivariate analysis. Consequently, the multivariate analysis showed that TNM stage (HR = 2.282; 95%CI: 1.547–3.365; *p* < 0.001) and TT (HR = 0.586; 95%CI: 0.404–0.849; *p* = 0.005) were independent prognostic indicators of ESCC survival. Thus, the TT before therapy may serve as a novel independent prognostic factor for ESCC.

**Table 3:** Univariate and multivariate cox hazards analysis for overall survival in 204 patients with ESCC.

	Univariate analysis			Multivariate analysis		
Variables	HR	95%CI	*p* value**	HR	95%CI	*p* value**
Gender						
Male vs. Female	0.762	0.503–1.155	0.762	0.990	0.641–1.528	0.962
Age (years)						
<59 vs.≥59	1.058	0.735–1.522	0.763	1.166	0.809–1.682	0.410
Histological differentiation*						
Differentiated vs. Undifferentiated	1.306	0.892–1.911	0.170			
T classification						
T3-4 vs. T1-2	2.288	1.412–3.709	0.001			
N classification						
Yes vs. No	2.158	1.491–3.124	0.000			
Metastasis						
Yes vs. No	2.543	1.393–4,460	0.002			
TNM stage^♯^						
III-IV vs. I-II	2.108	1.459–3.046	0.000	2.282	1.547–3.365	0.000
Tobacco history						
Yes vs. No	1.245	0.849–1.826	0.262			
PT						
≥12.0 vs. <12.0	0.988	0.686–1.422	0.948			
PTA%						
≥102.8vs. <102.8	1.267	0.880–1.826	0.203			
INR						
≥0.96 vs.<0.96	0.807	0.561–1.162	0.249			
APTT						
≥30.6 vs.<30.6	1.291	0.896–1.861	0.171			
fibrinogen						
≥4.00 vs.<4.00	1.165	0.790–1.717	0.441			
TT						
≥17.2 vs. <17.2	0.658	0.457–0.948	0.025	0.586	0.404–0.849	0.005
PLT						
≥400 vs.<400	0.985	0.624–1.555	0.948			

HR, Hazard ratio; 95% CI, 95% confidence interval; PT, Prothrombin Time; PTA%, Prothrombin Time Activity; INR, International Normalized Ratio; APTT, Activated Partial Thromboplastin Time; TT, Thrombin Time.

*Moderately and well differentiated histologic types were classified as differentiated carcinoma; Low levels of differentiation were defined as undifferentiated carcinoma.

**Cox hazard regression model.

^♯^TNM denotes tumor-node-metastasis.

**Fig 2:**
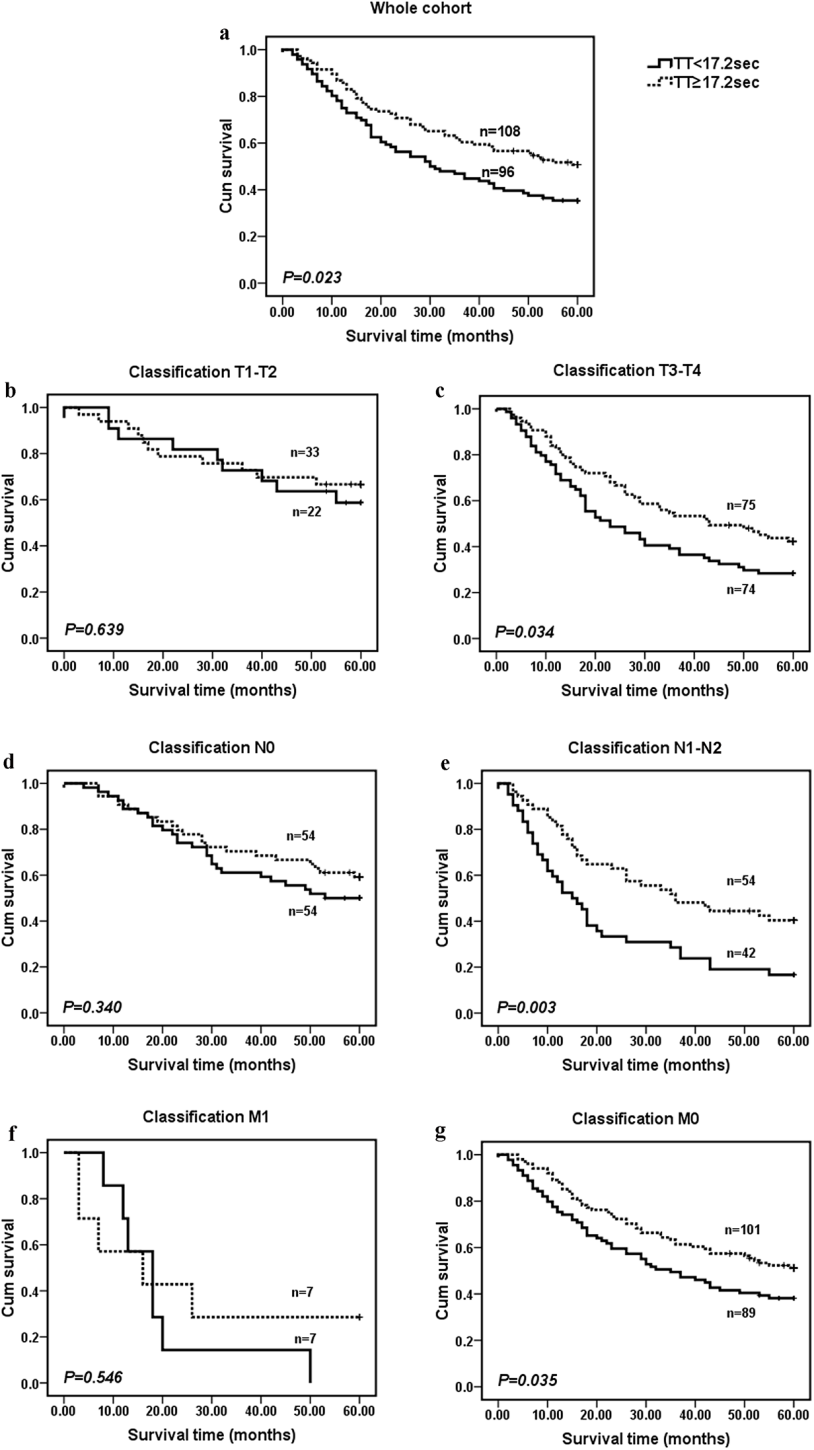
Prognostic significance of serum TT in ESCC. The patients were categorized into a low TT group and a normal TT group according to the media value (17.2 sec). The five-year overall survival rate was calculated using the Kaplan-Meier method and analyzed with the log-rank test. A high TT level was a favorable prognostic factor in the entire ESCC cohort (a), T3–T4 subgroup (c), N1–N2 subgroup (e), and M0 subgroup (g).

To further explore the prognostic significance of TT in ESCC, Kaplan-Meier survival curves were generated, and the groups were compared using the log-rank test. In the entire ESCC cohort, patients with a normal TT (n = 108) showed significantly better 5-year overall survival than patients with a lower TT (n = 96). The cumulative 5-year survival rate in the normal TT group was 46.3%, whereas it was only 32.3% in the lower TT group (*p* = 0.023, Fig 2A). We also analyzed the prognostic value of the TT in selective patient subgroups stratified according to the T classification, N classification, or metastasis. ESCC patients with a lower TT level exhibited significantly shorter overall survival than patients with a normal TT level in the T3-T4 subgroup (n = 149, *p* = 0.034, Fig 2C), N1 subgroup (n = 96, *p* = 0.003, Fig 2E), and M0 subgroup (n = 190, *p* = 0.035, Fig 2G).

### Relationship between the TT and the clinical characteristics in 204 patients with ESCC

The associations between the TT and clinical characteristics of the patients are summarized in Table 4. The TT was not associated with gender, age, tobacco history, histological differentiation, N classification, or metastasis. Compared with normal patients, the TT was lower and the fibrinogen level, T stage and TNM stage were higher in the entire ESCC cohort (*p* = 0.042, *p* = 0.024 and *p* = 0.029, respectively). Furthermore, the patient cohort was divided into two groups: the normal TT group and lower TT group, according to the mean TT (17.2 sec). Gender, age, histological differentiation, T classification, N classification, metastasis and TNM stage were similar between the two groups. However, patients without a tobacco history (*p* = 0.011) were more common in the normal TT group than in the lower TT group. Furthermore, patients with a lower TT were inclined to suffer hyperfibrinogenemia compared with the normal group (*p* = 0.005).

**Table 4:** Relationship between the TT and the clinical characteristics in 204 patients with ESCC.

	Cases	TT (sec)	*p* value*	Lower TT	Normal TT	*p* value**
Variables	(n)	(Mean ± SD)		<17.2(sec)	≥17.2(sec)	
Number of cases	204			96	108	
Gender						
Male	145	17.3±1.16	0.535	73	72	0.140
Female	59	17.2±1.03		23	36	
Age (≥59)						
No	101	17.2±1.27	0.308	53	48	0.125
Yes	103	17.3±0.95		43	60	
Tobacco history						
No	76	17.3±0.96	0.479	27	49	***0*.*011***
Yes	128	17.2±1.20		69	59	
fibrinogen (≥4.0 g/L)						
No	141	17.3±1.06	***0*.*042***	57	84	***0*.*005***
Yes	63	17.0±1.22		39	24	
Histological differentiation						
Poor	64	17.1±1.08	0.562	27	37	0.380
Moderate	95	17.3±1.13		44	51	
Well	45	17.3±1.14		25	20	
T classification						
T1	16	17.8±1.37	***0*.*024***	5	11	0.360
T2	38	17.4±1.07		17	21	
T3	124	17.1±1.06		64	60	
T4	25	17.4±1.16		10	15	
Tis	1	19.1			1	
N classification						
No	108	17.1±1.11	0.093	54	54	0.372
Yes	96	17.4±1.12		42	54	
Metastasis						
No	190	17.2±1.11	0.426	89	101	0.819
Yes	14	17.5±1.23		7	7	
TNM stage^♯^						
I	15	18.0±1.39	***0*.*029***	4	11	0.078
II	97	17.1±1.06		52	45	
III	81	17.3±1.06		33	48	
IV	11	17.3±1.09		7	4	

Bold italics indicate significant differences (*p* < 0.05). Mean ± SD, Mean ± standard deviation; TT, Thrombin Time.

*P values were calculated using unpaired Student’s t-tests or one-way ANOVA, *p* <0.05 indicated significant differences.

**P values were calculated using the chi-squared test (χ^2^ test), *p* <0.05 indicated significant differences.

^♯^TNM denotes tumor-node-metastasis.

## Discussion

Our results show that the PT, APTT, and fibrinogen levels were significantly higher and the TT was lower in patients with ESCC compared with normal controls. Previous studies indicated that the most common coagulation abnormalities related to malignancy are a mild shortening or prolongation of the PT or APTT, increased levels of fibrinogen degradation products (FDP) and fibrin-specific D-dimer fragments (DD), as a result of fibrinolytic response to activated coagulation, and elevated fibrinogen levels [21–22]. Although coagulation tests can be measured in the laboratory, patients may be asymptomatic. Furthermore, little is known about the abnormalities and prognostic value of coagulation tests in patients with ESCC, despite the identification of associations between serum coagulation markers and some cancers [13–20]. Our study revealed that of the seven coagulation parameters (PT, PTA, INR, APTT, fibrinogen, TT and PLT), only the TT was a prognostic indicator for ESCC. In addition, we found that patients with a lower TT experienced significantly shorter overall survival than patients with a normal TT, not only in the entire cohort but also in the subgroups stratified by T stage (T3-T4), N stage (N1-2), and metastasis (M0).

The TT test is a simple, inexpensive test that is widely used in clinical laboratories to detect the function of coagulation, anticoagulation and fibrinolysis. Both the extrinsic and intrinsic coagulation cascades culminate in the activation of prothrombin to thrombin and the subsequent formation of a fibrin clot. Simultaneously, the fibrinolysis system is activated and fibrin is lysed. Thrombosis and disseminated intravascular coagulation (DIC) are common complications in cancer. The hallmark of DIC is an excessive release of thrombin and the subsequent formation and deposition of fibrin in the vessels of several organs. However, the pathogenesis of the prothrombotic state in cancer is more complex and multifactorial and is involved in tumor-procoagulant activity, the host inflammatory response and cancer treatment, but the associated mechanisms are not entirely understood [23]. Many tumors produce procoagulant substances, such as tissue factor (TF) and cancer procoagulant (CP), that can either directly or indirectly activate the blood clotting cascade by inducing a an inflammatory response in the patient. This inflammatory response then provides feedback to the tumor to release additional procoagulant factors [24–27]. Moreover, the release of potent inflammatory mediators, such as TNF and IL-1, from activated macrophages and stimulated T-cells can further enhance the prothrombotic process [24]. The suppression of fibrinolytic activity and/or decrease in anticoagulant factors can also promote the coagulation of cancer. Thromboembolic complications have been observed in cancer patients irrespective of the levels of anticoagulant factors, indicating that these levels are unfortunately not predictive of thrombotic disease [28].

Thrombin leads to the formation of fibrin, which accumulates in cancer tissue and acts as a protective barrier against inflammatory cells [29]. The prolongation of the TT decreases the level of fibrinogen or alters its structure and results in overactive fibrinolysis in some conditions, such as DIC, fibrinogenopenia, heparin anticoagulant therapy and cancer. In our study, the TT was lower and the fibrinogen level was higher in patients with ESCC compared with normal controls (17.2 sec vs.18.6 sec; 3.44 g/L vs. 2.89 g/L; *p* < 0.001 for both). Furthermore, patients with moderate or advanced disease exhibited higher fibrinogen levels than patients with early-stage disease (Table 2). In addition, a low TT was indicative of hyperfibrinogenemia (*p* = 0.005). These findings indicate that moderate to advanced ECSS is associated with a lower TT and a higher fibrinogen level, which lead to shorter overall survival. The lack of differences between T1-2, N0 and TT patients was attributed to the fact that all 204 patients were asymptomatic. Moreover, the TT remained in the normal range and only slightly changed when patients reached the moderate or advanced disease stage. Lastly, the number of M1-2 cases was insufficient for the analysis.

In this study, the incidence of hyperfibrinogenemia was 30.9%(63/204, cut-off value 4.0 g/L), which is lower than that reported in other ESCC studies (43.7%) [30]. Moreover, hyperfibrinogenemia was found to be positively correlated with tumor length, depth of invasion, pathological stage and disease recurrence in ESCC [30], and the plasma fibrinogen level was identified as a predictive marker for the postoperative recurrence of ESCC in patients receiving neoadjuvant treatment [31]. Elevated plasma fibrinogen levels have also been shown to correlate with decreased OS in univariate and multivariate analyses [32], and patients with advanced disease exhibited higher plasma fibrinogen levels than patients with early-stage disease [33]. Accordingly, patients with moderate or advanced disease exhibited higher fibrinogen levels than patients with early-stage disease in the current study. Fibrinogen is a dimeric molecule with multiple integrin and non-integrin binding motifs, and cancer cells often express high levels of integrins or intercellular adhesion molecule 1. Fibrinogen deposition around tumor cells enhances the interaction between these cells and platelets, which effectively form microemboli in target organs [34]. Fibrinogen layers help tumor cells block natural killer cytotoxicity with thrombin, which can protect tumor cells from the innate immune system [35]. Thus, the clinical consequences of hyperfibrinogenemia can be serious and negatively impact the course of the disease.

In summary, we first analyzed the clinical significance of pretreatment plasma coagulation tests in 204 ESCC patients treated with surgery. In this study, the TT was an independent prognostic factor in ESCC, both in the entire cohort and in groups stratified by T classification, N classification and metastasis. Prognostic markers for ESCC are currently lacking, and the TT test is a highly reproducible assay that can easily be conducted in all diagnostic laboratories. Thus, it may be employed as a prognostic tool for patients with ESCC in conjunction with other markers.

## References

[1] Jemal A, Bray F, Center MM, Ferlay J, Ward E, Forman D. Global cancer statistics. CA Cancer J Clin 2011;61(2):69–90. 10.3322/caac.20107 21296855

[2] Ke L. Mortality and incidence trends from esophagus cancer in selected geographic areas of China circa 1970–90. Int J Cancer 2002;102(3):271–4. 12397650 10.1002/ijc.10706

[3] Enzinger PC, Mayer RJ. Esophageal cancer. N Engl J Med 2003;349(23):2241–52. 14657432 10.1056/NEJMra035010

[4] Ilson DH, Wadleigh RG, Leichman LP, Kelsen DP. Paclitaxel given by a weekly 1-h infusion in advanced esophageal cancer. Ann Oncol 2007;18(5):898–902. 17351256 10.1093/annonc/mdm004

[5] Kuderer NM, Ortel TL, Francis CW. Impact of venous thromboembolism and anticoagulation on cancer and cancer survival. J Clin Oncol 2009;27(29):4902–11. 10.1200/JCO.2009.22.4584 19738120 PMC2799059

[6] Khorana AA, Francis CW, Culakova E, Fisher RI, Kuderer NM, Lyman GH. Thromboembolism in hospitalized neutropenic cancer patients. J Clin Oncol 2006;24(3):484–90. 16421425 10.1200/JCO.2005.03.8877

[7] Blom JW, Doggen CJ, Osanto S, Rosendaal FR. Malignancies, prothrombotic mutations, and the risk of venous thrombosis. JAMA 2005;293(6):715–22. 15701913 10.1001/jama.293.6.715

[8] Iversen LH, Thorlacius-Ussing O. Relationship of coagulation test abnormalities to tumour burden and postoperative DVT in resected colorectal cancer. Thromb Haemost 2002;87(3):402–8. 11916070

[9] Thodiyil PA, Kakkar AK. Variation in relative risk of venous thromboembolism in different cancers. Thromb Haemost 2002;87(6):1076–7. 12083490

[10] Amirkhosravi A, Meyer T, Amaya M, Davila M, Mousa SA, Robson T, et al The role of tissue factor pathway inhibitor in tumor growth and metastasis. Semin Thromb Hemost 2007;33(7):643–52. 18000790 10.1055/s-2007-991531

[11] Langer F, Holstein K, Eifrig B, Bokemeyer C. [Haemostatic aspects in clinical oncology]. Hamostaseologie 2008;28(5):472–80. 19132177

[12] Falanga A, Marchetti M, Vignoli A, Balducci D. Clotting mechanisms and cancer: implications in thrombus formation and tumor progression. Clin Adv Hematol Oncol 2003;1(11):673–8. 16258469

[13] Tas F, Kilic L, Serilmez M, Keskin S, Sen F, Duranyildiz D. Clinical and prognostic significance of coagulation assays in lung cancer. Respiratory Medicine 2013;107(3):451–457. 10.1016/j.rmed.2012.11.007 23200643

[14] Oya M, Akiyama Y, Okuyama T, Ishikawa H. High preoperative plasma D-dimer level is associated with advanced tumor stage and short survival after curative resection in patients with colorectal cancer. Jpn J Clin Oncol 2001;31(8):388–94. 11574632 10.1093/jjco/hye075

[15] Bottasso B, Mari D, Coppola R, Santoro N, Vaglini M, Mannucci PM. Hypercoagulability and hyperfibrinolysis in patients with melanoma. Thromb Res 1996;81(3):345–52. 8928092 10.1016/0049-3848(96)00006-0

[16] Oberhoff C, Rollwagen C, Tauchert AM, Hoffmann O, Winkler UH, Schindler AE. Perioperative development of a thrombogenic risk profile in patients with carcinomas of the breast: a cause of increased thrombosis. Eur J Gynaecol Oncol 2000;21(6):560–8. 11214610

[17] Sun W, Ren H, Gao C, Ma W, Luo L, Liu Y, et al Clinical and Prognostic Significance of Coagulation Assays in Pancreatic Cancer Patients With Absence of Venous Thromboembolism. American Journal of Clinical Oncology 2015:1. 24401666 10.1097/01.coc.0000436088.69084.22

[18] Shu YJ, Weng H, Bao RF, Wu XS, Ding Q, Cao Y, et al Clinical and prognostic significance of preoperative plasma hyperfibrinogenemia in gallbladder cancer patients following surgical resection: a retrospective and in vitro study. BMC Cancer 2014;14:566 10.1186/1471-2407-14-566 25096189 PMC4131047

[19] Mytnik M, Stasko J. D-dimer, plasminogen activator inhibitor-1, prothrombin fragments and protein C—role in prothrombotic state of colorectal cancer. Neoplasma 2011;58(3):235–8. 21391740 10.4149/neo_2011_03_235

[20] Dirix LY, Salgado R, Weytjens R, Colpaert C, Benoy I, Huget P, et al Plasma fibrin D-dimer levels correlate with tumour volume, progression rate and survival in patients with metastatic breast cancer. Br J Cancer 2002;86(3):389–95. 11875705 10.1038/sj.bjc.6600069 PMC2375200

[21] Goodnight SJ. Bleeding and intravascular clotting in malignancy: a review. Ann N Y Acad Sci 1974;230:271–88. 4522874 10.1111/j.1749-6632.1974.tb14459.x

[22] Rickles FR, Edwards RL. Activation of blood coagulation in cancer: Trousseau's syndrome revisited. Blood 1983;62(1):14–31. 6407544

[23] De Cicco M. The prothrombotic state in cancer: pathogenic mechanisms. Critical Reviews in Oncology/Hematology 2004;50(3):187–196. 15182825 10.1016/j.critrevonc.2003.10.003

[24] Falanga A, Rickles FR. Pathophysiology of the thrombophilic state in the cancer patient. Semin Thromb Hemost 1999;25(2):173–82. 10357085 10.1055/s-2007-994919

[25] Gouin-Thibault I, Achkar A, Samama MM. The thrombophilic state in cancer patients. Acta Haematol 2001;106(1–2):33–42. 11549775 10.1159/000046587

[26] Falanga A, Donati MB. Pathogenesis of thrombosis in patients with malignancy. Int J Hematol 2001;73(2):137–44. 11372723 10.1007/BF02981929

[27] Rickles FR, Falanga A. Molecular basis for the relationship between thrombosis and cancer. Thromb Res 2001;102(6):V215–24. 11516455 10.1016/s0049-3848(01)00285-7

[28] Loreto MF, De Martinis M, Corsi MP, Modesti M, Ginaldi L. Coagulation and cancer: implications for diagnosis and management. Pathol Oncol Res 2000;6(4):301–12. 11173665 10.1007/BF03187336

[29] Bick RL. Coagulation abnormalities in malignancy: a review. Semin Thromb Hemost 1992;18(4):353–72. 1470924 10.1055/s-2007-1002575

[30] Wang J, Liu H, Shao N, Tan B, Song Q, Jia Y, et al The clinical significance of preoperative plasma fibrinogen level and platelet count in resectable esophageal squamous cell carcinoma. World Journal of Surgical Oncology 2015;13(1). 10.1186/s12957-015-0543-4 PMC4408570 25896470

[31] Matsuda S, Takeuchi H, Fukuda K, Nakamura R, Takahashi T, Wada N, et al Clinical significance of plasma fibrinogen level as a predictive marker for postoperative recurrence of esophageal squamous cell carcinoma in patients receiving neoadjuvant treatment. Dis Esophagus 2014;27(7):654–61. 10.1111/dote.12115 23980622

[32] Thurner EM, Krenn-Pilko S, Langsenlehner U, Stojakovic T, Pichler M, Gerger A, et al The association of an elevated plasma fibrinogen level with cancer-specific and overall survival in prostate cancer patients. World J Urol 2014. 10.1007/s00345-014-1459-2 25475065

[33] Hefler-Frischmuth K, Lafleur J, Hefler L, Polterauer S, Seebacher V, Reinthaller A, et al Plasma fibrinogen levels in patients with benign and malignant ovarian tumors. Gynecol Oncol 2015;136(3):567–70. 10.1016/j.ygyno.2014.12.041 25576886

[34] Shimada H, Oohira G, Okazumi S, Matsubara H, Nabeya Y, Hayashi H, et al Thrombocytosis associated with poor prognosis in patients with esophageal carcinoma. J Am Coll Surg 2004;198(5):737–41. 15110807 10.1016/j.jamcollsurg.2004.01.022

[35] Zheng S, Shen J, Jiao Y, Liu Y, Zhang C, Wei M, et al Platelets and fibrinogen facilitate each other in protecting tumor cells from natural killer cytotoxicity. Cancer Sci 2009;100(5):859–65. 10.1111/j.1349-7006.2009.01115.x 19302289 PMC11158185

